# Differences in mental health status during the COVID-19 pandemic between patients undergoing in-center hemodialysis and peritoneal dialysis

**DOI:** 10.1007/s40620-023-01747-0

**Published:** 2023-08-22

**Authors:** Pim Bouwmans, Zeinab Skalli, Robin W. M. Vernooij, Marc H. Hemmelder, Wanda S. Konijn, Joy Lips, Janneke Mulder, Anna A. Bonenkamp, Brigit C. van Jaarsveld, Alferso C. Abrahams, the DOMESTICO study group, A. C. Abrahams, M. C. Verhaar, B. C. van Jaarsveld, F. W. Dekker, F. J. van Ittersum, W. Konijn, M. H. Hemmelder, M. A. G. J. ten Dam, A. van Eck van der Sluijs, E. Driehuis, A. A. Bonenkamp, T. S. van Lieshout, A. J. Roeterdink, P. B. Leurs, M. R. Korte, J. B. van der Net, A. M. Schrander-vd Meer, T. T. Cnossen, B. C. van Jaarsveld, G. F. van Breda, A. De Vriese, J. Lips, H. P. Krepel, M. A. G. J. ten Dam, C. J. A. M. Konings, A. van Eck van der Sluijs, A. Lips, A. Özyilmaz, A. Neradova, F. T. J. Boereboom, S. van Esch, C. R. Susanto, E. J. Hoorn, D. Severs, A. H. Boonstra, R. W. Nette, M. A. M. Verhoeven, Y. M. Vermeeren, D. H. T. IJpelaar, N. H. Hommes, M. van Buren, J. M. Hofstra, K. W. Mui, S. H. Binnenmars, S. H. A. Diepeveen, E. K. Hoogeveen, T. Cornelis, S. Boorsma, J. I. Rotmans, A. M. van Alphen, E. J. R. Litjens, M. H. Hemmelder, W. M. T. Janssen, A. Kuijper, C. H. Beerenhout, L. Bierma, A. Y. Adema, R. M. J. Wijering, W. Rüger, R. J. Bosma, E. L. Penne, C. W. H. de Fijter, H. F. H. Brulez, H. W. van Hamersvelt, W. A. G. van der Meijden, S. J. Huisman, J. C. Verhave, G. van Kempen, H. H. T. I. Klein, C. E. Douma, W. J. W. Bos, J. D. Snoep, J. Mulder, C. F. M. Franssen, A. C. Abrahams, K. François, A. J. Luik, R. J. L. Klaassen, A. van Tellingen, M. M. G. Dekker, A. G. Weenink, M. M. E. Krekels

**Affiliations:** 1 Divsion of Nephrology, Department of Internal Medicine, Maastricht University Medical Center, Maastricht, The Netherlands; 2 CARIM School for Cardiovascular Diseases, University of Maastricht, Maastricht, The Netherlands; 3 Department of Nephrology and Hypertension, University Medical Center Utrecht, Utrecht, The Netherlands; 4 Julius Centre for Health Sciences and Primary Care, University Medical Centre Utrecht, Utrecht University, Utrecht, The Netherlands; 5 Dutch Kidney Patients Association (NVN), Bussum, The Netherlands; 6 Department of Internal Medicine, Bernhoven Hospital, Uden, The Netherlands; 7 Department of Internal Medicine, Treant Zorggroep, Emmen, The Netherlands; 8 Department of Nephrology, Amsterdam UMC Location Vrije Universiteit Amsterdam, Amsterdam, The Netherlands; 9 Amsterdam Cardiovascular Sciences, Diabetes and Metabolism, Amsterdam, The Netherlands; 10 Diapriva Dialysis Center, Amsterdam, The Netherlands

**Keywords:** COVID-19, Hemodialysis, Peritoneal dialysis, Quality of life, Cohort study, Pandemic

## Abstract

**Background:**

The mental health of dialysis patients during the COVID-19 pandemic may have been modulated by dialysis modality. Studies comparing mental health of in-center hemodialysis and peritoneal dialysis patients during the first 2 years of the pandemic are lacking.

**Methods:**

We conducted repeated cross-sectional and multivariable regression analyses to compare the mental health of in-center hemodialysis and peritoneal dialysis patients from March 2019 until August 2021 using data from the Dutch nOcturnal and hoME dialysis Study To Improve Clinical Outcomes. The study period was divided into one pre-pandemic and six 3-month pandemic periods (period 1–period 6). Mental health was assessed with the Mental Component Summary score of the 12-item Short Form health survey and mental symptoms of the Dialysis Symptom Index.

**Results:**

We included 1274 patients (968 on in-center hemodialysis and 306 on peritoneal dialysis). Mental Component Summary scores did not differ between in-center hemodialysis and peritoneal dialysis patients. In contrast, in-center hemodialysis patients more often reported nervousness during period 3 (27% vs 15%, *P* = 0.04), irritability and anxiety during period 3 (31% vs 18%, *P* = 0.03, 26% vs. 9%, *P* = 0.002, respectively) and period 4 (34% vs 22%, *P* = 0.04, 22% vs 11%, *P* = 0.03, respectively), and sadness in period 4 (38% vs 26%, *P* = 0.04) and period 5 (37% vs 22%, *P* = 0.009). Dialysis modality was independently associated with mental symptoms.

**Conclusions:**

In-center hemodialysis patients more often experienced mental symptoms compared to peritoneal dialysis patients from September 2020 to June 2021, which corresponds to the second lockdown of the COVID-19 pandemic. Mental health-related quality-of-life did not differ between in-center hemodialysis and peritoneal dialysis patients.

**Trial registration number:**

Netherlands Trial Register NL6519, date of registration: 22 August, 2017.

**Graphical abstract:**

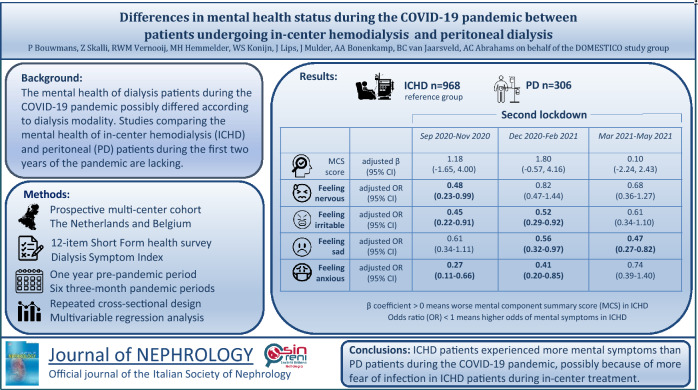

**Supplementary Information:**

The online version contains supplementary material available at 10.1007/s40620-023-01747-0.

## Introduction

The COVID-19 pandemic had a major impact on the mental health of the general population [1]. During lockdowns, nationwide restrictions mainly focused on protecting the general population against COVID-19. The combination of fear of COVID-19 and the impact of these restrictions may have impaired mental health, especially in patients with chronic diseases [2, 3]. Dialysis patients were also at risk of severe COVID-19 [4, 5], yet no changes in mental health were observed during the first COVID-19 wave [6]. Also during the second wave, from January 2021 to June 2021, no change in mental health was observed [7]. Since later phases of the pandemic were not included in these studies, it remains unknown to what extent the mental health of dialysis patients changed during the COVID-19 pandemic.

Only few studies have compared the mental health between in-center hemodialysis (ICHD) and peritoneal dialysis (PD) patients during the pandemic. In two Asian single-center, cross-sectional studies, ICHD patients experienced more psychological distress compared to PD patients during the first year of the pandemic [8, 9]. In contrast, PD patients more often reported anxiety in a Turkish cross-sectional multi-center study [10]. The poorer mental health among ICHD patients could be explained by difficulty to self-isolate during multiple dialysis center visits per week. This was not the case for PD patients who performed their dialysis treatment at home. Consequently, PD patients had less contact with other patients and dialysis staff [11], which could have caused feelings of loneliness. Differences in mental health between ICHD and PD patients are therefore likely to have occurred during the COVID-19 pandemic, however this has not been investigated extensively.

In this study, we aimed to assess whether dialysis modalities affected mental health differently during the first two years of the COVID-19 pandemic, including lockdown periods and the first COVID-19 vaccination campaign. It is important to identify possible differences in mental health between dialysis patients, so that appropriate support may be provided during future outbreaks. We hypothesized that ICHD patients experienced more anxiety because of fear of being infected with COVID-19 whilst visiting dialysis centers. We also expected PD patients to experience worse mental health due to long-lasting social isolation.

## Materials and methods

### Study population, design, and period

Adult patients participating in the Dutch nOcturnal and hoME dialysis Study To Improve Clinical Outcomes (DOMESTICO) were included for analysis. DOMESTICO is an ongoing multi-center prospective observational cohort study in the Netherlands and Belgium, comparing health-related quality of life (HRQOL), clinical outcomes, and cost-effectiveness between ICHD and home-treated dialysis patients. The study protocol was published previously [12]. Patient inclusion started on December 20th, 2017. Data were collected at baseline, 3 months, 6 months, and every 6 months thereafter and will end in December 2023. Primary ethical approval was obtained from the medical research ethics committee of the VU University Medical Center Amsterdam, on December 7th, 2017. The DOMESTICO study is registered in the International Clinical Trial Registry Platform (number: NTR6736, date of registration: 22 August, 2017).

**Fig. 1:**
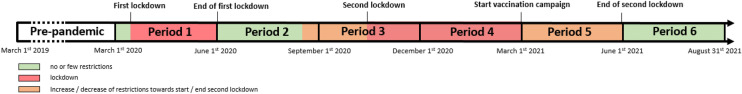
Overview of study periods in relation to restrictions and relaxations during the COVID-19 pandemic in the Netherlands

Using DOMESTICO data, we conducted repeated cross-sectional analyses to assess differences in mental health between ICHD and PD patients from the start of the COVID-19 pandemic on March 1st, 2020 to August 31st, 2021, 3 months after the end of the second lockdown. During the pandemic, COVID-19 incidence and restrictions varied widely. We therefore chose to divide the pandemic into six 3-month periods (P1–P6), as demonstrated in Fig. 1. This enabled us to distinguish characteristic phases of the pandemic, and include a representative sample in all periods. The pre-pandemic period, lasting from March 1st, 2019 to February 29th, 2020, was used as reference. Patients were included for analyses if they filled in at least one questionnaire in one of the above mentioned periods. We excluded home hemodialysis patients from analysis because of low representation in the cohort (*n* = 14, see Figure S2). We reported our results in accordance with the Strengthening the Reporting of Observational Studies in Epidemiology (STROBE) reporting guidelines (see Supplement Materials) [13].

### Data collection

Mental HRQOL was assessed using the Mental Component Summary (MCS) score of the 12-item Short Form (SF-12) health survey. Mental Component Summary scores were calculated using standard algorithms, meaning that a healthy individual scores 50 points on a scale of 0–100 with a standard deviation of 10 points. Mental symptoms were measured using the Dialysis Symptom Index (DSI), from which we included 8 mental symptoms, namely difficulty concentrating, worrying, feeling nervous, difficulty falling asleep, difficulty staying asleep, feeling irritable, feeling sad, and feeling anxious. Symptom severity was assessed on a 5-point Likert Scale (1 “no burden”, 2 “some burden”, 3 “moderate burden”, 4 “much burden”, and 5 “severe burden”).

Primary kidney disease was categorized according to the European Renal Association (ERA) coding system [14]. Comorbidity was assessed by the Charlson Comorbidity Index (CCI) [15]. Patients were categorized into three CCI categories (CCI score of 2 reflecting only end-stage kidney disease; CCI score of 3–4 reflecting intermediate comorbidity; CCI score of ≥ 5 reflecting severe comorbidity). Psychiatric treatment was defined as active treatment for psychiatric disease at baseline. We additionally retrieved data on COVID-19 vaccination from the Dutch Dialysis Registry (RENINE). Living situation was categorized as “Living alone” or “Living with other”. Educational level was categorized into “Higher”, “Intermediate” or “Lower” according to Statistics Netherlands [16]. Participants’ employment status was derived from the iMTA Productivity Cost Questionnaire (iPCQ). Employment was categorized into “Yes”, “No, unemployed or unfit to work” or “No, retired, other or unknown reason”.

### Statistical analyses

Patient characteristics were presented at baseline and periodically by using descriptive statistics. Baseline was set at time of study inclusion in DOMESTICO. Mental health was evaluated periodically for all patients first, and subsequently stratified for ICHD and PD patients. We compared MCS scores between ICHD and PD patients using the Student’s *t* test, and used Chi-squared test to compare the prevalence of mental symptoms. We further tested for differences in mental symptom severity (1–2 versus 3–5) by using Chi-squared test or Fisher’s exact test if assumptions were violated.

Next, we performed multivariable regression analyses for associations between dialysis modality and mental health. Linear regression analysis was used for MCS scores, and logistic regression for mental symptoms. Confounders were selected on theoretical plausibility, and clustered in four different models. In model 1, we introduced age, and sex. Subsequently, we introduced psychiatric treatment in model 2, acute start of dialysis treatment in model 3, and employment status and living situation in model 4. Estimates were presented as adjusted odds ratio’s (aOR). Regression analyses were performed on an imputed dataset. For imputation, we used multiple imputation by chained equations (MICE) with 50 iterations to construct five imputed datasets. We only imputed variables that were identified as confounders. As a sensitivity analysis, we repeated regression analyses on the non-imputed dataset. All analyses were performed using IBM SPSS Statistics 27. A significance level (*α*) < 0.05 was considered statistically significant.

## Results

### Study population and patient characteristics

Of the 1993 dialysis patients enrolled in DOMESTICO, 1274 filled in a questionnaire during the study period, of whom 968 were ICHD and 306 were PD patients (Figure S1). At baseline, ICHD and PD patients were aged 65 ± 14 and 64 ± 14 years, respectively (Table 1). The majority of patients were male (ICHD: 68%, PD: 60%). Peritoneal dialysis patients had fewer additional comorbidities compared to ICHD patients (CCI score of 2: 38% versus 29%). Dialysis treatment more often started acutely in ICHD than in PD (20% versus 5%), and the median dialysis vintage was longer in ICHD (11 months [4–17]) than in PD patients (6 months [2–17]). Among dialysis patients whose SARS-CoV-2 vaccination status was known (*n* = 649, 51%), 28 patients (4%) refused vaccination.

**Table 1:** Baseline characteristics of in-center hemodialysis and peritoneal dialysis patients

	ICHD	PD
	*n* = 968	*n* = 306
Age, years, mean (SD)	65 (14)	64 (14)
Sex, male, *n* (%)	654 (68)	185 (60)
Primary kidney disease, *n* (%)		
Glomerular disease	92 (10)	30 (10)
Pyelonephritis	42 (4)	14 (5)
Polycystic kidney disease	48 (5)	17 (6)
Hypertension	123 (13)	35 (11)
Renal vascular disease	71 (7)	30 (10)
Diabetic kidney disease	149 (15)	41 (13)
Miscellaneous	127 (13)	40 (13)
Unknown	316 (33)	99 (32)
Charlson Comorbidity Index (CCI), *n* (%)		
CCI 2	281 (29)	115 (38)
CCI 3–4	364 (38)	103 (34)
CCI ≥ 5	282 (29)	73 (24)
Unknown	41 (4)	15 (5)
BMI^a^, kg/m^2^, mean (SD)	28 (6)	27 (5)
Psychiatric treatment, *n* (%)	17 (2)	10 (3)
Acute start dialysis, *n* (%)	192 (20)	14 (5)
Dialysis vintage, months, median (IQR)	11 (4–17)	6 (2–17)
Previous kidney transplantation, *n* (%)		
Yes	88 (9)	22 (7)
No	552 (57)	166 (54)
Unknown	328 (34)	118 (39)
Living situation, *n* (%)		
Living alone	272 (28)	77 (25)
Living with others	571 (59)	191 (62)
Unknown	125 (13)	38 (12)
Educational level, *n* (%)		
Higher	182 (19)	51 (17)
Intermediate	195 (20)	72 (24)
Lower	410 (42)	127 (42)
Other	51 (5)	14 (5)
Unknown	130 (13)	42 (14)
Employment, *n* (%)		
Yes	137 (14)	56 (18)
No, unemployed or unfit for work	220 (23)	58 (19)
No, retired, other or unknown reason	596 (62)	191 (62)
Unknown	15 (2)	1 (< 1)
COVID-19 vaccination, *n* (%)		
Vaccinated	479 (49)	142 (46)
Not vaccinated	21 (2)	7 (2)
Unknown	468 (48)	157 (51)

*ICHD* in-center hemodialysis, *PD* peritoneal dialysis, *SD* standard deviation, *BMI* body mass index, *IQR* interquartile range

^a^698 dialysis patients (55%) have data available on BMI

Inclusion of patients in the six different periods during the COVID-19 pandemic is shown in Figure S1. The characteristics of ICHD and PD patients within each period are summarized in Table S1. Periodically, we found a similar distribution of sex and comorbidity as present at baseline. In-center hemodialysis patients had a higher median dialysis vintage than PD patients in P5 and P6 (12 [4–19] vs 8 [3–19] and 12 [5–19] vs 7 [3–19] months, respectively). In-center hemodialysis patients more frequently were unemployed or unfit for work compared to PD patients (P3: 21% vs 13%, P4: 21% vs 15%, P5: 23% vs 11%, respectively), and more often lived alone (P1: 30% vs 25%, P5: 31% vs 21%).

### Mental health in dialysis patients

**Fig. 2:**
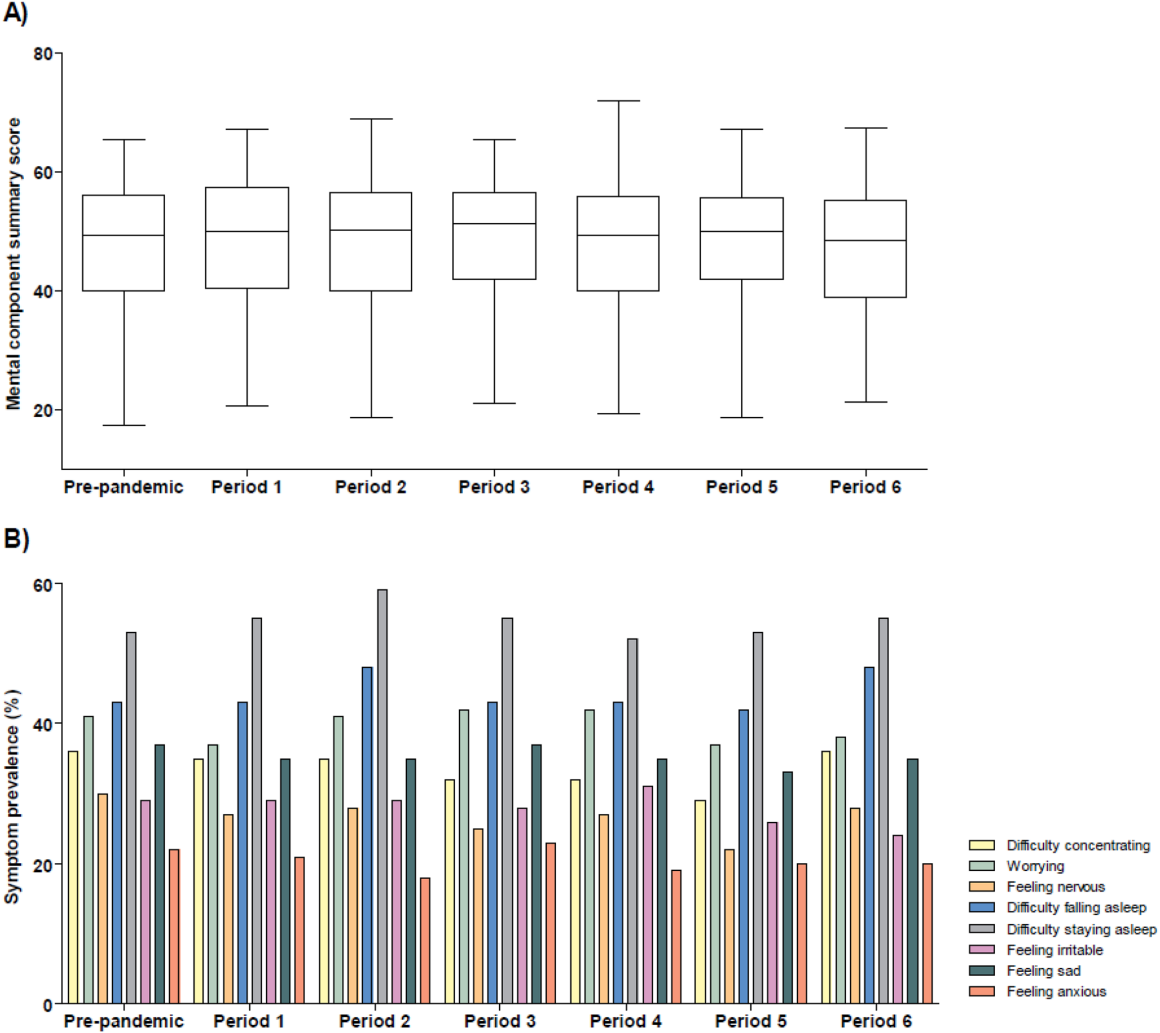
Mental component summary score (**A**) and prevalence of mental symptoms (**B**) in dialysis patients before and during the COVID-19 pandemic

The mean MCS score of all dialysis patients during any study period, varied between 47 and 49 (Fig. 2 and Table S2). Sleeping problems and worrying were the most reported mental symptoms in all periods (difficulty staying asleep in 52–59%; difficulty falling asleep in 42–48%; worrying 37–42%). Less frequently reported symptoms during the pandemic were sadness (33–37%), difficulty concentrating (29–36%), irritability (24–31%), nervousness (22–30%), and anxiety (18–23%).

**Fig. 3:**
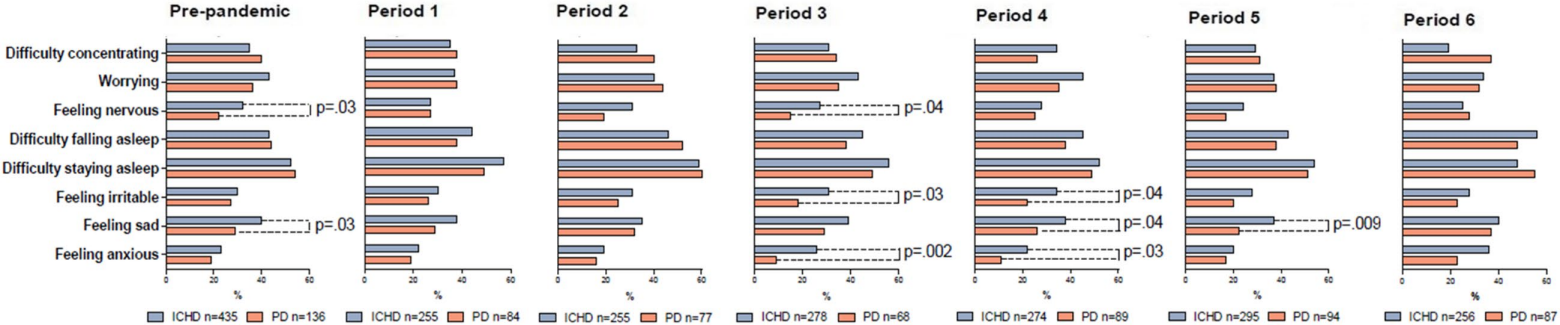
Prevalence of mental symptoms among ICHD and PD patients before and during the COVID-19 pandemic

The mental health status in ICHD and PD patients before and during the pandemic is presented in Table S3. The mean MCS score did not differ between ICHD and PD patients during any period. Before the pandemic, nervousness and sadness were more prevalent in ICHD patients than in PD patients (32% vs 22%, *p* = 0.03, and 40% vs 29%, *p* = 0.03, respectively), as shown in Fig. 3. These differences were not found in P1 and P2. During P3, however, more ICHD patients reported feeling nervous compared to PD patients (27% vs. 15%, *p* = 0.04), and more often felt sad in P4 (38% vs 26%, *p* = 0.04) and in P5 (37% vs 22%, *p* = 0.009). In contrast to nervousness and sadness, we observed differences in irritability and anxiety that were not present before the pandemic. In P3 and P4, ICHD patients more often reported feeling irritable (P3: 31% vs 18%, *p* = 0.03, P4: 34% vs 22%, *p* = 0.04), or anxious (P3: 26% vs 9%, *p* = 0.002, P4: 22% vs 11%, *p* = 0.03). These differences in mental symptoms were not accompanied by differences in symptom severity (Table S4).

**Fig. 4:**
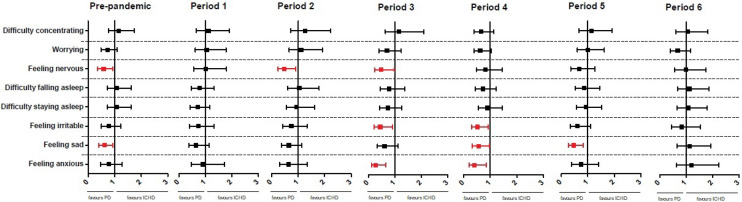
Adjusted Odds Ratios for mental symptoms of in-center hemodialysis and peritoneal dialysis patients before and during the COVID-19 pandemic. Adjusted for age, sex, psychiatric treatment, acute start dialysis, living situation and employment status

In multivariable regression analysis, dialysis modality was independently associated with mental symptoms (Fig. 4, Table S5). In comparison to ICHD patients, we found a lower prevalence in PD patients for anxiety in P3 (aOR 0.27 [0.11–0.66], *p* = 0.004) and P4 (aOR 0.41 [0.20–0.85], *p* = 0.02), and irritability in P3 (aOR 0.45 [0.22–0.91], *p* = 0.03) and P4 (aOR 0.52 [0.29–0.92], *p* = 0.03). These associations were not observed before the COVID-19 pandemic. In contrast, both before and during the pandemic, PD was associated with lower prevalence of nervousness (pre-pandemic: aOR 0.58 [0.36–0.92], *p* = 0.02, P2: aOR 0.47 [0.24–0.91], *p* = 0.03, P3: 0.48 [0.23–0.99], *p* = 0.05) and sadness (pre-pandemic: aOR 0.61 [0.39–0.93], *p* = 0.02, P4: aOR 0.56 [0.32–0.97], *p* = 0.04, P5: 0.47 [0.27–0.82], *p* = 0.008). We did not find any association between dialysis modality and MCS scores. Sensitivity analysis, using the non-imputed dataset, showed similar results (Table S6).

## Discussion

In this study, we found a higher prevalence of mental symptoms in ICHD compared to PD patients in the period September 2020 to June 2021, which corresponds to the second lockdown. In-center hemodialysis patients reported more nervousness, sadness, irritability, and anxiety compared to PD patients. Interestingly, these differences were not reflected by a lower mental HRQOL in ICHD patients.

Previous studies reported differences in mental health between ICHD and PD patients during the COVID-19 pandemic. Two single-center cross sectional studies found higher mental burden in ICHD patients. A Chinese study used the Impact of Event Scale (IES), and found higher pandemic-related stress levels in 76 ICHD patients compared to 156 PD patients [8]. A Korean study surveyed 148 dialysis patients during a COVID-19 incidence peak, and found higher scores of depression, anxiety, and stress in ICHD patients [9]. In contrast, a Turkish multi-center cross-sectional study involving 116 ICHD and 130 PD patients found a higher prevalence of anxiety in PD patients [10], measured by the Hospital Anxiety and Depression Scale (HADS). Our study measured mental health in a large cohort during multiple periods of the COVID-19 pandemic. In contrast to what we expected, mental HRQOL was not lower in PD than in ICHD patients, despite social isolation, lack of peer support and less contact with healthcare providers. We found a higher prevalence of mental symptoms in ICHD compared to PD patients, which might suggest that ICHD patients felt unsafe in dialysis centers during the pandemic.

Differences in mental symptoms between ICHD and PD patients could be explained by differences between treatment locations. Peritoneal dialysis patients were preferably contacted by telephone by the treatment center, and could more easily self-isolate during lockdowns. In ICHD patients, fear of contracting COVID-19 during group patient transfers or dialysis treatment has previously been related to increased anxiety [17, 18]. These concerns seem reasonable given the high COVID-19-related mortality rate in this population at that time [4, 5, 19]. In dialysis centers, preventive measures included distancing, wearing facemasks, temperature controls and isolation if COVID-19 was suspected. In addition, ICHD patients experienced other patients being severely ill or dying from COVID-19. These experiences could have frequently confronted ICHD patients with the possibility and consequences of being infected. Additional measures, such as targeted psychosocial support or information provision could be considered to decrease the impact of mental stressors present within dialysis centers [17, 20, 21]. However, these supporting care facilities were not always available during the pandemic. Our findings justify considering additional measures to preserve mental health during future pandemic outbreaks, especially in ICHD patients, and demonstrate the advantages of home-based dialysis treatment.

During the pandemic, differences in mental symptoms between ICHD and PD patients were solely observed in study periods corresponding to the second lockdown in the Netherlands (P3–P5), but disappeared after COVID-19 vaccination (P6). After commencement of the vaccination program, mental health between ICHD and PD no longer differed. Almost all patients in our cohort whose vaccination status was known were vaccinated (96%). Within the Dutch dialysis population, high antibody levels have been measured after COVID-19 vaccination [22], which has been correlated with lower risk of severe COVID-19 [23, 24]. These findings were shared with dialysis staff and patients, which could have decreased concerns. Previous data showed that anxiety and depression scores in dialysis patients decreased 15 days after vaccination [21]. In our cohort, vaccination might have also decreased fear of COVID-19.

We observed no difference in mental HRQOL between ICHD and PD patients, despite higher rates of mental symptoms in ICHD patients. These contradictory findings suggest lack of construct validity of MCS scores to detect differences in mental health. On the other hand, the SF-12 questionnaire has previously demonstrated adequate responsiveness in measuring mental HRQOL when compared to the 36-item Short Form health survey (SF-36) [25]. Clinically relevant change, however, has not yet been established for SF-12 outcomes in dialysis patients. An advantage of the DSI is that symptoms are measured on a 5-point Likert scale. A one-unit-increment on the scale is likely to represent a clinically relevant change, as has been demonstrated for another questionnaire [26]. In this study, we demonstrate the utility of using both a generic HRQOL and a symptom questionnaire when aiming to measure differences in mental health. Future research should address construct validity and minimal import change for the SF-12 health survey in dialysis patients.

The repeated cross-sectional design in our study is not suitable to analyze changes in mental health over time, but other studies were able to do so. Two single-center Chinese studies of 247 and 100 ICHD patients found lower HRQOL and higher rates of anxiety during the first lockdown, respectively [17, 27]. A previous DOMESTICO cohort study, by Bonenkamp et al., found similar results in 177 Dutch dialysis patients during the first lockdown [6]. In another Dutch study, Nadort et al. found no difference in anxiety and depression in 121 ICHD patients during the second lockdown [7]. The stable mental health observed in Dutch dialysis patients was explained by a high resilience against mental stressors. In addition, the majority of dialysis patients were unemployed, and their weekly routine consisted of frequent dialysis treatments. Dialysis patients might have therefore experienced only limited impact of nation-wide restrictions on daily life.

Our study has some limitations. We did not conduct longitudinal analysis on the mental health in our cohort because only a small proportion filled in a questionnaire before and during at least two pandemic periods involving restrictions. Given the prevalence of mental symptoms in our cohort, we would have not been able to correct for relevant confounders in order to assess an independent association between dialysis modality and mental health. By performing a repeated cross-sectional analysis, we were able to demonstrate this independent association during different periods of the COVID-19 pandemic. This type of analysis has previously been used to study mental health during the pandemic [28, 29]. Second, we have chosen a three-month interval to categorize the COVID-19 pandemic in separate periods. We consider this interval to adequately capture the rapidly changing circumstances during the pandemic. Although these intervals do not completely match the key characteristic periods, the overlap is substantial and therefore suitable to distinguish these phases from one another. Third, we did not collect data on COVID-19 diagnosis, and could therefore not assess to what extent COVID-19 explains the observed difference in mental symptoms between ICHD and PD patients. In another ongoing Dutch prospective observational cohort study, data on COVID-19 diagnosis in dialysis patients is being collected [30]. This study might address the possible impact of COVID-19 diagnosis on mental health in dialysis patients.

The strength of our study lies within the sample size, data collection, and the follow-up during the different periods in the first two pandemic years. First, we included a large population of 1274 dialysis patients from centers in the Netherlands and Belgium, which enhances external validity of our findings. Second, we prospectively collected granular individual data. Third, we were able to include reference periods both before the pandemic, and after the end of the second lockdown. Fourth, data was complete for the majority of cases, and sensitivity analysis on non-imputed data showed comparable results.

In conclusion, ICHD patients experienced more mental symptoms than PD patients during the COVID-19 pandemic, which endured as long as restrictive measures were applied. Monitoring mental health in dialysis patients during future pandemics is of importance to adequately support those who might benefit from additional supportive measures.

### Supplementary Information

Below is the link to the electronic supplementary material.

Supplementary file1 (DOCX 213 KB)

## Data Availability

The datasets generated during and/or analyzed during the current study are subject to an embargo of twelve months after completion of the DOMESTICO study. Once the embargo expires, the data will be available upon reasonable request.
