# Supplementary material for: Differences in mental health status during the COVID-19 pandemic between patients undergoing in-center hemodialysis and peritoneal dialysis
Source: J Nephrol. 2023 Aug 22;36(7):2037–46. doi: 10.1007/s40620-023-01747-0 (PMC10543747; doi:10.1007/s40620-023-01747-0)
Supplement: Supplementary file 1 — Supplementary file1 (DOCX 213 KB) [file 40620_2023_1747_MOESM1_ESM.docx]

**Supplementary Table 1. Patient characteristics according to dialysis modality before and during the COVID-19 pandemic .**

|  | **Pre-pandemic** | | **Period 1** | | **Period 2** | | **Period 3** | | **Period 4** | | **Period 5** | | **Period 6** | | |  |
| --- | --- | --- | --- | --- | --- | --- | --- | --- | --- | --- | --- | --- | --- | --- | --- | --- |
|  | 1^st^ March 2019 –  28^th^ February 2020 | | 1^st^ March 2020 –  31^st^ May 2020 | | 1^st^ June 2020 –  31^st^ August 2020 | | 1^st^ September 2020 –  30^th^ November 2020 | | 1^st^ December 2020 –  29^th^ February 2021 | | 1^st^ March 2021 –  31^st^ May 2021 | | 1^st^ June 2021 –  31^st^ August 2021 | | |  |
|  | **ICHD** | **PD** | **ICHD** | **PD** | **ICHD** | **PD** | **ICHD** | **PD** | **ICHD** | **PD** | **ICHD** | **PD** | | **ICHD** | **PD** |  |
| Patient included for analysis | 440 | 136 | 264 | 84 | 260 | 77 | 281 | 69 | 280 | 89 | 300 | 95 | | 266 | 90 |  |
| Age, years, mean (SD) | 64 (14) | 64 (13) | 65 (13) | 65 (13) | 67 (13) | 66 (15) | 66 (13) | 68 (12) | 66 (14) | 66 (13) | 66 (13) | 65 (12) | | 67 (13) | 65 (13) |  |
| Sex, male, n (%) | 298 (68) | 86 (63) | 176 (67) | 54 (64) | 168 (65) | 40 (52) | 195 (69) | 37 (54) | 182 (65) | 53 (60) | 214 (71) | 60 (63) | | 183 (69) | 55 (61) |  |
| Primary kidney disease, n (%) |  |  |  |  |  |  |  |  |  |  |  |  | |  |  |  |
| - Glomerular disease | 58 (13) | 17 (13) | 30 (11) | 11 (13) | 28 (11) | 8 (10) | 32 (11) | 6 (9) | 25 (9) | 6 (7) | 25 (8) | 7 (7) | | 18 (7) | 1 (1) |  |
| - Pyelonephritis | 20 (5) | 7 (5) | 13 (5) | 2 (2) | 14 (5) | 2 (3) | 13 (5) | 2 (3) | 14 (5) | 5 (6) | 13 (4) | 4 (4) | | 9 (3) | 5 (6) |  |
| - Polycystic kidney disease | 22 (5) | 9 (7) | 17 (6) | 8 (10) | 16 (6) | 3 (4) | 25 (9) | 6 (9) | 17 (6) | 6 (7) | 12 (4) | 7 (7) | | 10 (4) | 3 (3) |  |
| - Hypertension | 65 (15) | 17 (13) | 34 (13) | 14 (17) | 39 (15) | 11 (14) | 40 (14) | 12 (17) | 46 (16) | 11 (12) | 36 (12) | 12 (13) | | 25 (9) | 8 (9) |  |
| - Renal vascular disease | 42 (10) | 17 (13) | 27 (10) | 12 (14) | 26 (10) | 13 (17) | 22 (8) | 9 (13) | 27 (10) | 11 (12) | 16 (5) | 12 (13) | | 15 (6) | 6 (7) |  |
| - Diabetic kidney disease | 83 (19) | 23 (17) | 53 (20) | 11 (13) | 35 (13) | 13 (17) | 45 (16) | 11 (16) | 44 (16) | 15 (17) | 42 (14) | 8 (8) | | 26 (10) | 8 (9) |  |
| - Miscellaneous | 71 (16) | 21 (15) | 39 (15) | 11 (13) | 48 (19) | 15 (19) | 42 (15) | 8 (12) | 41 (15) | 13 (15) | 33 (11) | 10 (11) | | 25 (9) | 10 (11) |  |
| - Unknown | 79 (18) | 25 (18) | 51 (19) | 15 (18) | 54 (21) | 12 (16) | 62 (22) | 15 (22) | 66 (24) | 22 (25) | 123 (41) | 35 (37) | | 138 (52) | 49 (54) |  |
| BMI, kg/m^2^, mean (SD) | 28 (6) | 27 (4) | 29 (6) | 27 (5) | 27 (5) | 26 (4) | 28 (5) | 26 (4) | 27 (5) | 27 (4) | 28 (5) | 26 (5) | | 27 (5) | 27 (5) |  |
| Charlson Comorbidity index, n (%) |  |  |  |  |  |  |  |  |  |  |  |  | |  |  |  |
| - CCI 2 | 136 (31) | 56 (41) | 77 (29) | 37 (44) | 80 (31) | 30 (39) | 91 (32) | 25 (36) | 74 (26) | 37 (42) | 74 (24) | 41 (43) | | 73 (27) | 30 (33) |  |
| - CCI 3-4 | 161 (37) | 37 (27) | 96 (36) | 26 (31) | 108 (42) | 27 (35) | 105 (37) | 25 (36) | 105 (38) | 28 (31) | 117 (39) | 33 (35) | | 104 (39) | 36 (40) |  |
| - CCI ≥ 5 | 133 (30) | 35 (26) | 88 (33) | 19 (23) | 68 (26) | 17 (22) | 82 (29) | 19 (28) | 88 (31) | 22 (25) | 95 (32) | 17 (18) | | 76 (29) | 19 (21) |  |
| - Unknown | 10 (2) | 8 (6) | 3 (1) | 2 (2) | 4 (2) | 3 (4) | 3 (1) | - | 13 (5) | 2 (2) | 14 (5) | 4 (4) | | 13 (5) | 5 (6) |  |
| Psychiatric treatment, n (%) | 6 (1) | 5 (4) | 3 (1) | 3 (4) | 3 (1) | 6 (8) | 5 (2) | 2 (3) | 6 (2) | 2 (2) | 6 (2) | - | | 2 (<1) | 4 (4) |  |
| Acute start dialysis, n (%) | 84 (19) | 15 (11) | 55 (21) | 5 (6) | 46 (18) | 5 (6) | 57 (20) | 6 (9) | 41 (15) | 6 (7) | 56 (19) | 5 (5) | | 42 (16) | 6 (7) |  |
| Dialysis vintage, months, median (IQR) | 7 (4-11) | 8 (4-11) | 6 (4-12) | 5 (3-12) | 6 (3-12) | 7 (4-13) | 8 (4-14) | 7 (4-14) | 8 (4-14) | 7 (3-13) | 12 (4-19) | 8 (3-19) | | 12 (5-19) | 7 (3-19) |  |
| Previous kidney transplantation, n (%) |  |  |  |  |  |  |  |  |  |  |  |  | |  |  |  |
| - Yes | 48 (11) | 11 (8) | 23 (9) | 8 (10) | 21 (8) | 3 (4) | 27 (10) | 5 (7) | 30 (11) | 5 (6) | 22 (7) | 7 (7) | | 15 (6) | 6 (7) |  |
| - No | 302 (69) | 93 (68) | 190 (72) | 57 (68) | 191 (73) | 54 (70) | 181 (64) | 46 (67) | 176 (63) | 57 (64) | 151 (50) | 48 (51) | | 113 (42) | 30 (33) |  |
| - Unknown | 90 (20) | 32 (24) | 51 (19) | 19 (23) | 48 (18) | 20 (26) | 73 (26) | 18 (26) | 74 (26) | 27 (30) | 127 (42) | 40 (42) | | 138 (52) | 54 (60) |  |
| Living situation, n (%) |  |  |  |  |  |  |  |  |  |  |  |  | |  |  |  |
| - Living alone | 120 (27) | 35 (26) | 80 (30) | 21 (25) | 80 (31) | 21 (27) | 76 (27) | 17 (25) | 77 (28) | 21 (24) | 92 (31) | 20 (21) | | 83 (31) | 27 (30) |  |
| - Living with others | 277 (63) | 84 (62) | 155 (59) | 52 (62) | 159 (61) | 47 (61) | 174 (62) | 47 (68) | 181 (65) | 56 (63) | 165 (55) | 65 (68) | | 149 (56) | 53 (59) |  |
| - Unknown | 43 (10) | 17 (13) | 29 (11) | 11 (13) | 21 (8) | 9 (12) | 31 (11) | 5 (7) | 22 (8) | 12 (13) | 43 (14) | 10 (11) | | 34 (13) | 10 (11) |  |
| Educational level, n (%) |  |  |  |  |  |  |  |  |  |  |  |  | |  |  |  |
| - Higher | 85 (19) | 27 (20) | 44 (17) | 14 (17) | 42 (16) | 9 (12) | 59 (21) | 15 (22) | 58 (21) | 12 (13) | 63 (21) | 18 (19) | | 52 (20) | 14 (16) |  |
| - Intermediate | 91 (21) | 29 (21) | 60 (23) | 19 (23) | 51 (20) | 19 (25) | 63 (22) | 16 (23) | 51 (18) | 21 (24) | 65 (22) | 19 (20) | | 44 (17) | 20 (22) |  |
| - Lower | 192 (44) | 59 (43) | 116 (44) | 35 (42) | 132 (51) | 36 (47) | 113 (40) | 30 (43) | 133 (48) | 41 (46) | 112 (37) | 41 (43) | | 122 (46) | 39 (43) |  |
| - Other | 26 (6) | 4 (3) | 9 (3) | 5 (6) | 11 (4) | 2 (3) | 13 (5) | 2 (3) | 14 (5) | 1 (1) | 18 (6) | 5 (5) | | 13 (5) | 7 (8) |  |
| - Unknown | 46 (10) | 17 (13) | 35 (13) | 11 (13) | 24 (9) | 11 (14) | 33 (12) | 6 (9) | 24 (9) | 14 (16) | 42 (14) | 12 (13) | | 35 (13) | 10 (11) |  |
| Employment, n (%) |  |  |  |  |  |  |  |  |  |  |  |  | |  |  |  |
| - Yes | 72 (16) | 28 (21) | 39 (15) | 14 (17) | 37 (14) | 11 (14) | 39 (14) | 7 (10) | 30 (11) | 17 (19) | 41 (14) | 23 (24) | | 36 (14) | 20 (22) |  |
| - No, unemployed or unfit for work | 101 (23) | 27 (20) | 54 (20) | 14 (17) | 46 (18) | 15 (20) | 60 (21) | 9 (13) | 60 (21) | 13 (15) | 68 (23) | 10 (11) | | 52 (20) | 18 (20) |  |
| - No, retired, other or unknown reason | 257 (58) | 81 (60) | 171 (65) | 55 (65) | 177 (68) | 51 (66) | 181 (64) | 53 (77) | 190 (68) | 59 (66) | 189 (63) | 62 (65) | | 173 (65) | 52 (58) |  |
| - Unknown | 10 (2) | - | - | 1 (1) | - | - | 1 (<1) | - | - | - | 2 (1) | - | | 5 (2) | - |  |
| COVID-19 vaccination, n (%) |  |  |  |  |  |  |  |  |  |  |  |  | |  |  |  |
| - Vaccinated | - | - | - | - | - | - | - | - | 4 (1) | - | 181 (60) | 56 (59) | | 152 (57) | 44 (49) |  |
| - Not vaccinated | 202 (46) | 58 (43) | 134 (51) | 46 (55) | 139 (53) | 38 (49) | 175 (62) | 38 (55) | 176 (63) | 51 (57) | 13 (4) | 2 (2) | | 4 (2) | 1 (1) |  |
| - Unknown | 238 (54) | 78 (57) | 130 (49) | 38 (45) | 121 (47) | 39 (51) | 106 (38) | 31 (45) | 100 (36) | 38 (43) | 106 (35) | 37 (39) | | 110 (41) | 45 (50) |  |
| ICHD: in-center hemodialysis, PD: peritoneal dialysis, SD: standard deviation, BMI: body mass index, IQR: interquartile range. | | | | | | | | | | | | | | | | |

**Supplementary Table 2. Mental health of dialysis patients before and during the COVID-19 pandemic.**

|  | **Pre-pandemic** | **Period 1** | **Period 2** | **Period 3** | **Period 4** | **Period 5** | **Period 6** |
| --- | --- | --- | --- | --- | --- | --- | --- |
|  | 1^st^ March 2019 –  28^th^ February 2020 | 1^st^ March 2020 –  31^st^ May 2020 | 1^st^ June 2020 –  31^st^ August 2020 | 1^st^ September 2020 –  30^th^ November 2020 | 1^st^ December 2020 –  29^th^ February 2021 | 1^st^ March 2021 –  31^st^ May 2021 | 1^st^ June 2021 –  31^st^ August 2021 |
| **SF-12** | **n = 512** | **n = 306** | **n = 297** | **n = 301** | **n = 330** | **n = 347** | **n = 320** |
| MCS score, mean (SD) | 48 (10) | 49 (10) | 48 (10) | 49 (10) | 48 (10) | 48 (9) | 47 (10) |
| **Dialysis symptom index** | **n = 571** | **n = 339** | **n = 332** | **n = 346** | **n = 363** | **n = 389** | **n = 343** |
| Difficulty concentrating, n (%) | 208 (36) | 120 (35) | 115 (35) | 111 (32) | 116 (32) | 114 (29) | 123 (36) |
| Worrying, n (%) | 235 (41) | 127 (37) | 136 (41) | 144 (42) | 153 (42) | 145 (37) | 130 (38) |
| Feeling nervous, n (%) | 169 (30) | 92 (27) | 93 (28) | 85 (25) | 98 (27) | 86 (22) | 95 (28) |
| Difficulty falling asleep, n (%) | 248 (43) | 145 (43) | 158 (48) | 150 (43) | 157 (43) | 162 (42) | 165 (48) |
| Difficulty staying asleep, n (%) | 301 (53) | 187 (55) | 196 (59) | 189 (55) | 187 (52) | 207 (53) | 190 (55) |
| Feeling irritable, n (%) | 166 (29) | 98 (29) | 97 (29) | 97 (28) | 114 (31) | 102 (26) | 84 (24) |
| Feeling sad, n (%) | 213 (37) | 120 (35) | 115 (35) | 129 (37) | 126 (35) | 130 (33) | 120 (35) |
| Feeling anxious, n (%) | 124 (22) | 72 (21) | 61 (18) | 79 (23) | 69 (19) | 76 (20) | 69 (20) |
| SF-12: 12-item short-form health survey, MCS: mental component summary, SD: standard deviation. | | | | | | | |

**Supplementary Table 3. Differences in mental health between in-center hemodialysis and peritoneal dialysis patients before and during the COVID-19 pandemic.**

|  | **Pre-pandemic** | | | **Period 1** | | | | **Period 2** | | | **Period 3** | | | **Period 4** | | | **Period 5** | | | **Period 6** | | |
| --- | --- | --- | --- | --- | --- | --- | --- | --- | --- | --- | --- | --- | --- | --- | --- | --- | --- | --- | --- | --- | --- | --- |
|  | 1^st^ March 2019 –  28^th^ February 2020 | | | 1^st^ March 2020 –  31^st^ May 2020 | | | | 1^st^ June 2020 –  31^st^ August 2020 | | | 1^st^ September 2020 –  30^th^ November 2020 | | | 1^st^ December 2020 –  29^th^ February 2021 | | | 1^st^ March 2021 –  31^st^ May 2021 | | | 1^st^ June 2021 –  31^st^ August 2021 | | |
|  | **ICHD** | **PD** | p | | **ICHD** | **PD** | p | **ICHD** | **PD** | p | **ICHD** | **PD** | p | **ICHD** | **PD** | p | **ICHD** | **PD** | p | **ICHD** | **PD** | p |
| **SF-12** | **n = 389** | **n = 123** |  | | **n = 231** | **n = 75** |  | **n = 229** | **n = 68** |  | **n = 242** | **n = 59** |  | **n = 248** | **n = 82** |  | **n = 257** | **n = 90** |  | **n = 240** | **n = 80** |  |
| MCS score, mean (SD) | 47 (10) | 49 (10) | .07 | | 48 (10) | 50 (11) | .4 | 48 (10) | 49 (9) | .6 | 48 (10) | 50 (8) | .2 | 48 (10) | 50 (9) | .1 | 48 (10) | 49 (9) | .7 | 48 (10) | 46 (10) | .1 |
| **Dialysis symptom index** | **n = 435** | **n = 136** |  | | **n = 255** | **n = 84** |  | **n = 255** | **n = 77** |  | **n = 278** | **n = 68** |  | **n = 274** | **n = 89** |  | **n = 295** | **n = 94** |  | **n = 256** | **n = 87** |  |
| Difficulty concentrating | 154 (35) | 54 (40) | .4 | | 88 (35) | 32 (38) | .6 | 84 (33) | 31 (40) | .2 | 88 (32) | 23 (34) | .7 | 93 (34) | 23 (26) | .2 | 85 (29) | 29 (31) | .7 | 91 (36) | 32 (37) | .8 |
| Worrying | 186 (43) | 49 (36) | .2 | | 95 (37) | 32 (38) | .9 | 102 (40) | 34 (44) | .5 | 120 (43) | 24 (35) | .2 | 122 (45) | 31 (35) | .1 | 109 (37) | 36 (38) | .8 | 102 (40) | 28 (32) | .2 |
| Feeling nervous | **139 (32)** | **30 (22)** | **.03** | | 69 (27) | 23 (27) | .9 | 78 (31) | 15 (19) | .06 | **75 (27)** | **10 (15)** | **.04** | 76 (28) | 22 (25) | .6 | 70 (24) | 16 (17) | .2 | 71 (28) | 24 (28) | .9 |
| Difficulty falling asleep | 188 (43) | 60 (44) | .9 | | 113 (44) | 32 (38) | .3 | 118 (46) | 40 (52) | .4 | 124 (45) | 26 (38) | .3 | 123 (45) | 34 (38) | .3 | 126 (43) | 36 (38) | .5 | 123 (48) | 42 (48) | .9 |
| Difficulty staying asleep | 227 (52) | 74 (54) | .7 | | 146 (57) | 41 (49) | .2 | 150 (59) | 46 (60) | .9 | 156 (56) | 33 (49) | .3 | 143 (52) | 44 (49) | .7 | 159 (54) | 48 (51) | .6 | 142 (55) | 48 (55) | .9 |
| Feeling irritable | 130 (30) | 36 (26) | .4 | | 76 (30) | 22 (26) | .5 | 78 (31) | 19 (25) | .3 | **85 (31)** | **12 (18)** | **.03** | **94 (34)** | **20 (22)** | **.04** | 83 (28) | 19 (20) | .1 | 64 (25) | 20 (23) | .7 |
| Feeling sad | **173 (40)** | **40 (29)** | **.03** | | 96 (38) | 24 (29) | .1 | 90 (35) | 25 (32) | .6 | 109 (39) | 20 (29) | .1 | **103 (38)** | **23 (26)** | **.04** | **109 (37)** | **21 (22)** | **.009** | 88 (34) | 32 (37) | .7 |
| Feeling anxious | 98 (23) | 26 (19) | .4 | | 56 (22) | 16 (19) | .6 | 49 (19) | 12 (16) | .5 | **73 (26)** | **6 (9)** | **.002** | **59 (22)** | **10 (11)** | **.03** | 60 (20) | 16 (17) | .6 | 49 (19) | 20 (23) | .4 |
| ICHD: in-center hemodialysis, PD: peritoneal dialysis, SF-12: 12-item short-form health survey, MCS: mental component summary, SD: standard deviation | | | | | | | | | | | | | | | | | | | | | | |

**Supplementary Table 4. Severity of mental symptom in ICHD and PD patient before and during the COVID-19 pandemic.**

|  | **Pre-pandemic** | | | **Period 1** | | | **Period 2** | | | **Period 3** | | | **Period 4** | | | **Period 5** | | | | **Period 6** | | |
| --- | --- | --- | --- | --- | --- | --- | --- | --- | --- | --- | --- | --- | --- | --- | --- | --- | --- | --- | --- | --- | --- | --- |
|  | 1^st^ March 2019 –  28^th^ February 2020 | | | 1^st^ March 2020 –  31^st^ May 2020 | | | 1^st^ June 2020 –  31^st^ August 2020 | | | 1^st^ September 2020 –  30^th^ November 2020 | | | 1^st^ December 2020 –  29^th^ February 2021 | | | 1^st^ March 2021 –  31^st^ May 2021 | | | | 1^st^ June 2021 –  31^st^ August 2021 | | |
|  | **ICHD** | **PD** | p | **ICHD** | **PD** | p | **ICHD** | **PD** | p | **ICHD** | **PD** | p | **ICHD** | **PD** | p | **ICHD** | **PD** | p | **ICHD** | | **PD** | p |
| **Difficulty concentrating** | **n = 147** | **n = 51** |  | **n = 86** | **n = 31** |  | **n = 84** | **n = 30** |  | **n = 87** | **n = 22** |  | **n = 92** | **n = 23** |  | **n = 84** | **n = 29** |  | **n = 89** | | **n = 32** |  |
| - ≤2 | 90 (61) | 32 (63) | .8 | 60 (70) | 22 (71) | .9 | 56 (67) | 16 (53) | .2 | 58 (67) | 11 (50) | .1 | 52 (57) | 14 (61) | .7 | 55 (65) | 20 (69) | .7 | 54 (61) | | 23 (72) | .3 |
| - ≥3 | 57 (39) | 19 (37) |  | 26 (30) | 9 (29) |  | 28 (33) | 14 (47) |  | 29 (33) | 11 (50) |  | 40 (43) | 9 (39) |  | 29 (35) | 9 (31) |  | 35 (39) | | 9 (28) |  |
| **Worrying** | **n = 179** | **n = 45** |  | **n = 92** | **n = 31** |  | **n = 98** | **n = 33** |  | **n = 118** | **n = 23** |  | **n = 119** | **n = 31** |  | **n = 105** | **n = 35** |  | **n = 102** | | **n = 28** |  |
| - ≤2 | 84 (47) | 22 (49) | .8 | 45 (49) | 16 (52) | .8 | 54 (55) | 22 (67) | .2 | 71 (60) | 16 (70) | .4 | 68 (57) | 18 (58) | .9 | 56 (53) | 25 (71) | .06 | 58 (57) | | 14 (50) | .5 |
| - ≥3 | 95 (53) | 23 (51) |  | 47 (51) | 15 (48) |  | 44 (45) | 11 (33) |  | 47 (40) | 7 (30) |  | 51 (43) | 13 (42) |  | 49 (47) | 10 (29) |  | 44 (43) | | 14 (50) |  |
| **Feeling nervous** | **n = 132** | **n = 29** |  | **n = 69** | **n = 23** |  | **n = 75** | **n = 15** |  | **n = 75** | **n = 10** |  | **n = 73** | **n = 22** |  | **n = 69** | **n = 16** |  | **n = 70** | | **n = 24** |  |
| - ≤2 | 83 (63) | 17 (59) | .7 | 42 (61) | 18 (78) | .1 | 53 (71) | 9 (60) | .5* | 44 (59) | 9 (90) | .08* | 43 (59) | 16 (73) | .2 | 43 (62) | 9 (56) | .7 | 42 (60) | | 15 (63) | .8 |
| - ≥3 | 49 (37) | 12 (41) |  | 27 (39) | 5 (22) |  | 22 (29) | 6 (40) |  | 31 (41) | 1 (10) |  | 30 (41) | 6 (27) |  | 26 (38) | 7 (44) |  | 28 (40) | | 9 (38) |  |
| **Difficulty falling asleep** | **n = 179** | **n = 58** |  | **n = 113** | **n = 31** |  | **n = 118** | **n = 40** |  | **n = 123** | **n = 26** |  | **n = 123** | **n = 34** |  | **n = 121** | **n = 35** |  | **n = 122** | | **n = 41** |  |
| - ≤2 | 73 (41) | 22 (38) | .7 | 43 (38) | 13 (42) | .7 | 49 (42) | 17 (43) | .9 | 55 (45) | 13 (50) | .6 | 48 (39) | 18 (53) | .1 | 47 (39) | 16 (46) | .5 | 45 (37) | | 17 (41) | .6 |
| - ≥3 | 106 (59) | 36 (62) |  | 70 (62) | 18 (58) |  | 69 (58) | 23 (58) |  | 68 (55) | 13 (50) |  | 75 (61) | 16 (47) |  | 74 (61) | 19 (54) |  | 77 (63) | | 24 (59) |  |
| **Difficulty staying asleep** | **n = 216** | **n = 72** |  | **n = 141** | **n = 41** |  | **n = 147** | **n = 46** |  | **n = 154** | **n = 30** |  | **n = 141** | **n = 42** |  | **n = 155** | **n = 44** |  | **n = 142** | | **n = 47** |  |
| - ≤2 | 82 (38) | 27 (38) | .9 | 59 (42) | 15 (37) | .5 | 59 (40) | 21 (46) | .5 | 68 (44) | 13 (43) | .9 | 54 (38) | 18 (43) | .6 | 56 (36) | 19 (43) | .4 | 49 (35) | | 24 (51) | .04 |
| - ≥3 | 134 (62) | 45 (63) |  | 82 (58) | 26 (63) |  | 88 (60) | 25 (54) |  | 86 (56) | 17 (57) |  | 87 (62) | 24 (57) |  | 99 (64) | 25 (57) |  | 93 (65) | | 23 (49) |  |
| **Feeling Irritable** | **n = 126** | **n = 34** |  | **n = 74** | **n = 22** |  | **n = 76** | **n = 18** |  | **n = 84** | **n = 12** |  | **n = 92** | **n = 20** |  | **n = 81** | **n = 19** |  | **n = 62** | | **n = 20** |  |
| - ≤2 | 74 (59) | 24 (71) | .2 | 47 (64) | 14 (64) | .9 | 53 (70) | 11 (61) | .5 | 54 (64) | 8 (67) | .9 | 56 (61) | 16 (80) | .1 | 51 (63) | 12 (63) | .9 | 36 (58) | | 11 (55) | .8 |
| - ≥3 | 52 (41) | 10 (29) |  | 27 (36) | 8 (36) |  | 23 (30) | 7 (39) |  | 30 (36) | 4 (33) |  | 36 (39) | 4 (20) |  | 30 (37) | 7 (37) |  | 26 (42) | | 9 (45) |  |
| **Feeling sad** | **n = 165** | **n = 37** |  | **n = 95** | **n = 23** |  | **n = 88** | **n = 25** |  | **n = 106** | **n = 19** |  | **n = 102** | **n = 23** |  | **n = 108** | **n = 21** |  | **n = 87** | | **n = 32** |  |
| - ≤2 | 110 (67) | 24 (65) | .8 | 50 (53) | 14 (61) | .5 | 58 (66) | 12 (48) | .1 | 65 (61) | 11 (58) | .8 | 62 (61) | 16 (70) | .4 | 67 (62) | 11 (52) | .4 | 55 (63) | | 20 (63) | .9 |
| - ≥3 | 55 (33) | 13 (35) |  | 45 (47) | 9 (39) |  | 30 (34) | 13 (52) |  | 41 (39) | 8 (42) |  | 40 (39) | 7 (30) |  | 41 (38) | 10 (48) |  | 32 (37) | | 12 (38) |  |
| **Feeling anxious** | **n = 92** | **n = 23** |  | **n = 55** | **n = 16** |  | **n = 47** | **n = 12** |  | **n = 72** | **n = 6** |  | **n = 58** | **n = 10** |  | **n = 58** | **n = 16** |  | **n = 49** | | **n = 20** |  |
| - ≤2 | 58 (63) | 12 (52) | .3 | 29 (53) | 13 (81) | .04 | 31 (66) | 9 (75) | .6* | 41 (57) | 6 (100) | .08 | 37 (64) | 7 (70) | .9 | 29 (50) | 9 (56) | .7 | 28 (57) | | 12 (60) | .8 |
| - ≥3 | 34 (37) | 11 (48) |  | 26 (47) | 3 (19) |  | 16 (34) | 3 (25) |  | 31 (43) | 0 (-) |  | 21 (36) | 3 (30) |  | 29 (50) | 7 (44) |  | 21 (43) | | 8 (40) |  |
| * Fisher’s exact test used because of violation of conditions for Chi squared test. Abbreviations: ICHD: in-center hemodialysis, PD: peritoneal dialysis. | | | | | | | | | | | | | | | | | | | | | | |

**Supplementary Table 5. Mental component summary score (β-coefficient [95% CI]) and mental symptoms (ORs [95% CI]) in peritoneal dialysis compared to in-center hemodialysis patients (imputed dataset).**

|  | **Pre-pandemic** | p | **Period 1** | p | **Period 2** | p | **Period 3** | p | **Period 4** | P | **Period 5** | p | **Period 6** | p |  |
| --- | --- | --- | --- | --- | --- | --- | --- | --- | --- | --- | --- | --- | --- | --- | --- |
|  | 1^st^ March 2019 –  28^th^ February 2020 |  | 1^st^ March 2020 –  31^st^ May 2020 |  | 1^st^ June 2020 –  31^st^ August 2020 |  | 1^st^ September 2020 –  30^th^ November 2020 |  | 1^st^ December 2020 –  29^th^ February 2021 |  | 1^st^ March 2021 –  31^st^ May 2021 |  | 1^st^ June 2021 –  31^st^ August 2021 |  |  |
| **Mental Component Summary score** | | | | | | | | | | | | | | | |
| 0 | 1.88 (-0.17, 3.93) | .07 | 1.20 (-1.48, 3.87) | .4 | 0.71 (-2.07, 3.49) | .6 | 1.15 (-1.66, 3.95) | .4 | 1.97 (-0.41, 4.34) | .1 | 0.49 (-1.79, 2.76) | .7 | -1.96 (-4.42, 0.50) | .1 |  |
| 1 | 1.94 (-0.11. 3.99) | .06 | 1.21 (-1.47, 3.88) | .4 | 0.98 (-1.80, 3.76) | .5 | 1.18 (-1.65, 4.01) | .4 | 2.02 (-0.31, 4.35) | .09 | 0.39 (-1.89, 2.67) | .7 | -1.73 (-4.20, 0.73) | .2 |  |
| 2 | 2.15 (0.11. 4.18) | .04 | 1.28 (-1.43, 4.00) | .4 | 1.63 (-1.17, 4.43) | .3 | 1.27 (-1.52, 4.07) | .4 | 2.02 (-0.31, 4.36) | .09 | 0.37 (-1.91, 2.65) | .7 | -1.58 (-4.06, 0.90) | .2 |  |
| 3 | 2.04 (0.00, 4.08) | .05 | 0.88 (-1.82, 3.59) | .5 | 1.06 (-1.72, 3.84) | .5 | 1.15 (-1.66, 3.96) | .4 | 1.86 (-0.48, 4.20) | .1 | 0.34 (-1.98, 2.65) | .8 | -1.67 (-4.18, 0.84) | .2 |  |
| 4 | 1.79 (-0.26, 3.84) | .09 | 0.76 (-1.97, 3.49) | .6 | 1.08 (-1.72, 3.87) | .5 | 1.18 (-1.65, 4.00) | .4 | 1.80 (-0.57, 4.16) | .1 | 0.10 (-2.24, 2.43) | .9 | -1.95 (-4.47, 0.57) | .1 |  |
| **Difficulty concentrating** | | | | | | | | | | | | | | | |
| 0 | 1.20 (0.81-1.79) | .4 | 1.17 (0.70-1.95) | .6 | 1.37 (0.81-2.32) | .2 | 1.10 (0.63-1.94) | .6 | 0.68 (0.40-1.16) | .2 | 1.10 (0.67-1.83) | .7 | 1.06 (0.64-1.75) | .8 |  |
| 1 | 1.20 (0.81-1.79) | .4 | 1.16 (0.70-1.95) | .6 | 1.35 (0.79-2.33) | .3 | 1.11 (0.62-1.98) | .7 | 0.67 (0.39-1.14) | .1 | 1.09 (0.65-1.81) | .7 | 0.97 (0.58-1.64) | .9 |  |
| 2 | 1.17 (0.79-1.75) | .4 | 1.13 (0.67-1.90) | .7 | 1.20 (0.69-2.11) | .5 | 1.09 (0.61-1.96) | .8 | 0.67 (0.39-1.14) | .1 | 1.11 (0.67-1.85) | .7 | 0.94 (0.55-1.59) | .8 |  |
| 3 | 1.18 (0.79-1.77) | .4 | 1.15 (0.68-1.95) | .6 | 1.28 (0.73-2.26) | .4 | 1.13 (0.63-2.03) | .7 | 0.66 (0.39-1.14) | .1 | 1.12 (0.67-1.89) | .7 | 1.00 (0.59-1.71) | .9 |  |
| 4 | 1.16 (0.77-1.74) | .5 | 1.12 (0.65-1.90) | .7 | 1.26 (0.71-2.23) | .4 | 1.16 (0.64-2.09) | .6 | 0.66 (0.38-1.14) | .1 | 1.13 (0.67-1.91) | .6 | 1.06 (0.62-1.82) | .8 |  |
| **Worrying** | | | | | | | | | | | | | | | |
| 0 | 0.75 (0.51-1.12) | .2 | 1.04 (0.62-1.72) | .9 | 1.19 (0.71-1.99) | .5 | 0.72 (0.41-1.25) | .2 | 0.67 (0.41-1.10) | .1 | 1.06 (0.66-1.71) | .8 | 0.72 (0.43-1.20) | .2 |  |
| 1 | 0.74 (0.49-1.10) | .1 | 1.03 (0.62-1.71) | .9 | 1.17 (0.69-1.97) | .6 | 0.69 (0.40-1.21) | .2 | 0.64 (0.39-1.06) | .08 | 1.05 (0.65-1.69) | .9 | 0.67 (0.40-1.13) | .1 |  |
| 2 | 0.71 (0.47-1.07) | .1 | 1.01 (0.61-1.70) | .9 | 1.06 (0.62-1.81) | .6 | 0.68 (0.38-1.19) | .2 | 0.64 (0.39-1.06) | .08 | 1.06 (0.65-1.71) | .8 | 0.66 (0.39-1.12) | .1 |  |
| 3 | 0.72 (0.48-1.08) | .1 | 1.09 (0.65-1.84) | .7 | 1.10 (0.64-1.89) | .7 | 0.69 (0.39-1.23) | .2 | 0.63 (0.38-1.05) | .08 | 0.99 (0.61-1.62) | .9 | 0.66 (0.39-1.13) | .1 |  |
| 4 | 0.73 (0.48-1.09) | .1 | 1.05 (0.62-1.78) | .9 | 1.11 (0.65-1.91) | .7 | 0.70 (0.40-1.24) | .2 | 0.62 (0.38-1.04) | .07 | 0.99 (0.60-1.62) | .9 | 0.68 (0.40-1.16) | .2 |  |
| **Feeling nervous** | | | | | | | | | | | | | | | |
| 0 | 0.60 (0.38-0.95) | .03 | 1.02 (0.58-1.77) | .9 | 0.55 (0.29-1.02) | .06 | 0.47 (0.23-0.96) | .04 | 0.86 (0.49-1.48) | .6 | 0.66 (0.36-1.20) | .2 | 0.98 (0.58-1.71) | .9 |  |
| 1 | 0.59 (0.38-0.93) | .02 | 1.01 (0.58-1.78) | .9 | 0.51 (0.27-0.96) | .04 | 0.46 (0.22-0.96) | .04 | 0.82 (0.47-1.44) | .5 | 0.63 (0.34-1.15) | .1 | 0.96 (0.56-1.67) | .9 |  |
| 2 | 0.57 (0.36-0.90) | .02 | 0.98 (0.56-1.73) | .9 | 0.46 (0.24-0.89) | .02 | 0.46 (0.22-0.96) | .04 | 0.82 (0.47-1.44) | .5 | 0.64 (0.35-1.19) | .2 | 0.92 (0.53-1.61) | .8 |  |
| 3 | 0.57 (0.36-0.90) | .02 | 1.00 (0.56-1.78) | .9 | 0.47 (0.24-0.90) | .02 | 0.47 (0.23-0.99) | .05 | 0.81 (0.46-1.41) | .4 | 0.64 (0.34-1.18) | .2 | 0.95 (0.54-1.66) | .9 |  |
| 4 | 0.58 (0.36-0.92) | .02 | 1.01 (0.56-1.80) | .9 | 0.47 (0.24-0.91) | .03 | 0.48 (0.23-0.99) | .05 | 0.82 (0.47-1.44) | .5 | 0.68 (0.36-1.27) | .2 | 0.99 (0.56-1.75) | .9 |  |
| **Difficulty falling asleep** | | | | | | | | | | | | | | | |
| 0 | 1.04 (0.70-1.53) | .9 | 0.77 (0.47-1.28) | .3 | 1.26 (0.75-2.09) | .4 | 0.77 (0.45-1.32) | .3 | 0.76 (0.47-1.24) | .3 | 0.83 (0.52-1.34) | .5 | 1.01 (0.62-1.64) | .9 |  |
| 1 | 1.03 (0.70-1.52) | .9 | 0.76 (0.46-1.27) | .3 | 1.19 (0.71-2.00) | .5 | 0.76 (0.44-1.33) | .3 | 0.73 (0.45-1.20) | .2 | 0.80 (0.49-1.29) | .4 | 0.99 (0.61-1.62) | .9 |  |
| 2 | 1.00 (0.68-1.48) | .9 | 0.76 (0.46-1.26) | .3 | 1.07 (0.63-1.82) | .8 | 0.76 (0.43-1.32) | .3 | 0.73 (0.45-1.20) | .2 | 0.80 (0.50-1.30) | .4 | 1.01 (0.62-1.66) | .9 |  |
| 3 | 1.05 (0.71-1.56) | .8 | 0.80 (0.48-1.35) | .4 | 1.04 (0.60-1.77) | .9 | 0.78 (0.44-1.36) | .4 | 0.71 (0.43-1.17) | .2 | 0.77 (0.48-1.26) | .3 | 1.05 (0.64-1.73) | .9 |  |
| 4 | 1.09 (0.73-1.62) | .7 | 0.79 (0.47-1.33) | .4 | 1.04 (0.61-1.79) | .9 | 0.78 (0.44-1.37) | .4 | 0.73 (0.44-1.21) | .2 | 0.87 (0.53-1.44) | .6 | 1.12 (0.68-1.85) | .7 |  |
| **Difficulty staying asleep** | | | | | | | | | | | | | | | |
| 0 | 1.09 (0.74-1.61) | .7 | 0.71 (0.43-1.17) | .2 | 1.04 (0.62-1.75) | .9 | 0.74 (0.43-1.25) | .3 | 0.90 (0.56-1.45) | .7 | 0.89 (0.56-1.42) | .6 | 0.99 (0.61-1.61) | .9 |  |
| 1 | 1.09 (0.74-1.60) | .7 | 0.71 (0.43-1.16) | .2 | 1.01 (0.60-1.71) | .9 | 0.73 (0.43-1.25) | .3 | 0.88 (0.54-1.42) | .6 | 0.88 (0.55-1.40) | .6 | 0.98 (0.60-1.60) | .9 |  |
| 2 | 1.06 (0.72-1.56) | .8 | 0.70 (0.43-1.15) | .2 | 0.92 (0.54-1.57) | .8 | 0.73 (0.42-1.25) | .2 | 0.88 (0.54-1.42) | .6 | 0.89 (0.56-1.41) | .6 | 0.98 (0.60-1.61) | .9 |  |
| 3 | 1.10 (0.74-1.64) | .6 | 0.72 (0.44-1.19) | .2 | 0.95 (0.55-1.63) | .8 | 0.73 (0.42-1.26) | .3 | 0.88 (0.54-1.43) | .6 | 0.94 (0.59-1.51) | .8 | 1.05 (0.64-1.74) | .8 |  |
| 4 | 1.09 (0.73-1.62) | .7 | 0.71 (0.43-1.17) | .2 | 0.94 (0.55-1.62) | .8 | 0.74 (0.43-1.27) | .3 | 0.89 (0.55-1.44) | .6 | 0.94 (0.58-1.52) | .8 | 1.09 (0.65-1.80) | .8 |  |
| **Feeling irritable** | | | | | | | | | | | | | | | |
| 0 | 0.85 (0.55-1.30) | .4 | 0.84 (0.48-1.46) | .5 | 0.74 (0.42-1.33) | .3 | 0.49 (0.25-0.95) | .04 | 0.56 (0.32-0.97) | .04 | 0.65 (0.37-1.14) | .1 | 0.90 (0.50-1.59) | .7 |  |
| 1 | 0.83 (0.54-1.29) | .4 | 0.84 (0.47-1.47) | .5 | 0.72 (0.40-1.30) | .3 | 0.48 (0.24-0.97) | .04 | 0.52 (0.29-0.92) | .02 | 0.63 (0.36-1.11) | .1 | 0.85 (0.47-1.51) | .6 |  |
| 2 | 0.79 (0.51-1.23) | .3 | 0.81 (0.46-1.43) | .5 | 0.61 (0.33-1.15) | .1 | 0.48 (0.24-0.96) | .04 | 0.52 (0.29-0.91) | .02 | 0.64 (0.36-1.15) | .1 | 0.80 (0.45-1.45) | .5 |  |
| 3 | 0.77 (0.49-1.20) | .2 | 0.80 (0.45-1.43) | .5 | 0.63 (0.34-1.17) | .1 | 0.47 (0.23-0.94) | .03 | 0.51 (0.29-0.91) | .02 | 0.58 (0.33-1.05) | .07 | 0.81 (0.44-1.46) | .5 |  |
| 4 | 0.78 (0.50-1.23) | .3 | 0.74 (0.41-1.33) | .3 | 0.61 (0.33-1.16) | .1 | 0.45 (0.22-0.91) | .03 | 0.52 (0.29-0.92) | .03 | 0.61 (0.34-1.10) | .1 | 0.84 (0.46-1.54) | .6 |  |
| **Feeling sad** | | | | | | | | | | | | | | | |
| 0 | 0.63 (0.42-0.96) | .03 | 0.66 (0.39-1.13) | .1 | 0.88 (0.51-1.52) | .6 | 0.65 (0.36-1.15) | .1 | 0.58 (0.34-0.99) | .05 | 0.49 (0.29-0.84) | .01 | 1.11 (0.67-1.84) | .7 |  |
| 1 | 0.60 (0.40-0.92) | .02 | 0.65 (0.38-1.12) | .1 | 0.79 (0.45-1.38) | .4 | 0.60 (0.33-1.08) | .09 | 0.54 (0.31-0.93) | .03 | 0.47 (0.27-0.81) | .006 | 1.05 (0.63-1.76) | .8 |  |
| 2 | 0.57 (0.37-0.88) | .01 | 0.64 (0.37-1.10) | .1 | 0.71 (0.40-1.25) | .2 | 0.59 (0.33-1.07) | .08 | 0.53 (0.31-0.92) | .03 | 0.47 (0.27-0.82) | .008 | 1.04 (0.62-1.75) | .9 |  |
| 3 | 0.59 (0.38-0.90) | .02 | 0.64 (0.37-1.11) | .1 | 0.75 (0.42-1.34) | .3 | 0.59 (0.33-1.08) | .09 | 0.55 (0.32-0.96) | .04 | 0.45 (0.26-0.79) | .005 | 1.07 (0.63-1.80) | .8 |  |
| 4 | 0.61 (0.39-0.93) | .02 | 0.65 (0.37-1.13) | .1 | 0.77 (0.43-1.38) | .4 | 0.61 (0.34-1.11) | .1 | 0.56 (0.32-0.97) | .04 | 0.47 (0.27-0.82) | .008 | 1.14 (0.67-1.93) | .7 |  |
| **Feeling anxious** | | | | | | | | | | | | | | | |
| 0 | 0.81 (0.50-1.32) | .4 | 0.84 (0.45-1.55) | .6 | 0.78 (0.39-1.55) | .5 | 0.27 (0.11-0.66) | .004 | 0.46 (0.23-0.95) | .04 | 0.80 (0.44-1.48) | .5 | 1.26 (0.70-2.27) | .4 |  |
| 1 | 0.79 (0.48-1.28) | .3 | 0.82 (0.44-1.54) | .5 | 0.72 (0.36-1.45) | .4 | 0.25 (0.10-0.60) | .002 | 0.43 (0.21-0.89) | .02 | 0.76 (0.41-1.40) | .4 | 1.19 (0.66-2.16) | .6 |  |
| 2 | 0.75 (0.46-1.23) | .3 | 0.80 (0.43-1.51) | .5 | 0.58 (0.27-1.23) | .2 | 0.25 (0.10-0.60) | .002 | 0.43 (0.21-0.89) | .02 | 0.79 (0.42-1.48) | .5 | 1.13 (0.62-2.08) | .7 |  |
| 3 | 0.76 (0.46-1.25) | .3 | 0.89 (0.47-1.70) | .7 | 0.63 (0.29-1.34) | .2 | 0.26 (0.10-0.63) | .003 | 0.42 (0.20-0.86) | .02 | 0.72 (0.38-1.35) | .3 | 1.08 (0.59-1.99) | .8 |  |
| 4 | 0.78 (0.47-1.28) | .3 | 0.90 (0.47-1.71) | .7 | 0.63 (0.29-1.34) | .2 | 0.27 (0.11-0.66) | .004 | 0.41 (0.20-0.85) | .02 | 0.74 (0.39-1.40) | .3 | 1.20 (0.64-2.23) | .6 |  |
| Model 0: crude analysis.  Model 1: age, sex.  Model 2: age, sex, psychiatric treatment.  Model 3: age, sex, psychiatric treatment, acute start dialysis.  Model 4: age, sex, psychiatric treatment, acute start dialysis, employment status, living situation. | | | | | | | | | | | | | | | |

**Supplementary Table 6. Mental component summary score (β-coefficient [95% CI]) and mental symptoms (ORs [95% CI]) in peritoneal dialysis compared to in-center hemodialysis patients (non-imputed dataset).**

|  | **Pre-pandemic** | p | **Period 1** | p | **Period 2** | p | **Period 3** | p | **Period 4** | p | **Period 5** | p | **Period 6** | p |
| --- | --- | --- | --- | --- | --- | --- | --- | --- | --- | --- | --- | --- | --- | --- |
|  | 1^st^ March 2019 –  28^th^ February 2020 |  | 1^st^ March 2020 –  31^st^ May 2020 |  | 1^st^ June 2020 –  31^st^ August 2020 |  | 1^st^ September 2020 –  30^th^ November 2020 |  | 1^st^ December 2020 –  29^th^ February 2021 |  | 1^st^ March 2021 –  31^st^ May 2021 |  | 1^st^ June 2021 –  31^st^ August 2021 |  |
| **Mental Component Score** | | | | | | | | | | | | | | |
| 0 | 1.88 (-0.18, 3.94) | .07 | 1.20 (-1.48, 3.87) | .4 | 0.71 (-2.07, 3.49) | .6 | 1.15 (-1.66, 3.95) | .4 | 1.97 (-0.41, 4.34) | .1 | 0.49 (-1.80, 2.77) | .7 | -1.96 (-4.43, 0.51) | .1 |
| 1 | 1.94 (-0.12. 3.99) | .06 | 1.21 (-1.47, 3.88) | .4 | 0.98 (-1.80, 3.76) | .5 | 1.18 (-1.65, 4.01) | .4 | 2.02 (-0.31, 4.35) | .09 | 0.39 (-1.89, 2.86) | .7 | -1.73 (-4.21, 0.74) | .2 |
| 2 | 2.32 (0.24. 4.39) | .03 | 1.28 (-1.43, 4.00) | .4 | 1.63 (-1.17, 4.43) | .3 | 1.27 (-1.52, 4.07) | .4 | 2.02 (-0.31, 4.36) | .09 | 0.52 (-1.81, 2.86) | .7 | -0.82 (-3.37, 1.73) | .5 |
| 3 | 2.19 (0.11, 4.27) | .04 | 0.86 (-1.88, 3.60) | .5 | 1.06 (-1.72, 3.84) | .5 | 1.15 (-1.66, 3.96) | .4 | 2.10 (-0.30, 4.51) | .09 | 0.50 (-1.87, 2.87) | .7 | -0.85 (-3.44, 1.73) | .5 |
| 4 | 1.85 (-0.38, 4.08) | .1 | 0.47 (-2.44, 3.39) | .8 | 1.08 (-1.72, 3.87) | .5 | 1.18 (-1.65, 4.00) | .4 | 1.39 (-1.22, 4.00) | .3 | 0.55 (-1.98, 3.09) | .7 | -1.02 (-3.76, 1.71) | .5 |
| **Difficulty concentrating** | | | | | | | | | | | | | | |
| 0 | 1.20 (0.81-1.79) | .4 | 1.17 (0.70-1.95) | .6 | 1.37 (0.81-2.32) | .2 | 1.10 (0.63-1.94) | .7 | 0.68 (0.40-1.16) | .2 | 1.10 (0.67-1.83) | .7 | 1.06 (0.64-1.75) | .8 |
| 1 | 1.20 (0.81-1.79) | .4 | 1.16 (0.70-1.95) | .6 | 1.35 (0.79-2.33) | .3 | 1.11 (0.62-1.98) | .7 | 0.67 (0.39-1.14) | .1 | 1.09 (0.65-1.81) | .7 | 0.97 (0.58-1.64) | .9 |
| 2 | 1.13 (0.75-1.70) | .6 | 1.09 (0.64-1.84) | .8 | 1.30 (0.74-2.28) | .4 | 1.12 (0.63-2.01) | .7 | 0.68 (0.39-1.16) | .2 | 1.07 (0.63-1.81) | .8 | 0.90 (0.53-1.54) | .7 |
| 3 | 1.14 (0.75-1.72) | .5 | 1.11 (0.65-1.89) | .7 | 1.38 (0.78-2.46) | .3 | 1.16 (0.65-2.09) | .5 | 0.67 (0.39-1.16) | .2 | 1.06 (0.62-1.81) | .8 | 0.96 (0.56-1.67) | .9 |
| 4 | 1.28 (0.83-1.99) | .3 | 1.26 (0.71-2.24) | .4 | 1.48 (0.81-2.70) | .2 | 1.36 (0.74-2.52) | .3 | 0.66 (0.37-1.20) | .2 | 0.98 (0.54-1.78) | .9 | 0.97 (0.54-1.73) | .9 |
| **Worrying** | | | | | | | | | | | | | | |
| 0 | 0.75 (0.51-1.12) | .2 | 1.04 (0.62-1.72) | .9 | 1.19 (0.71-1.99) | .5 | 0.72 (0.41-1.25) | .2 | 0.67 (0.41-1.10) | .1 | 1.06 (0.66-1.71) | .8 | 0.72 (0.43-1.20) | .2 |
| 1 | 0.74 (0.49-1.10) | .1 | 1.03 (0.62-1.71) | .9 | 1.17 (0.69-1.97) | .6 | 0.69 (0.40-1.21) | .2 | 0.64 (0.39-1.06) | .08 | 1.05 (0.65-1.69) | .9 | 0.67 (0.40-1.13) | .1 |
| 2 | 0.75 (0.50-1.14) | .2 | 0.98 (0.58-1.64) | .9 | 1.15 (0.67-1.98) | .6 | 0.69 (0.39-1.21) | .2 | 0.64 (0.38-1.07) | .09 | 1.04 (0.63-1.70) | .9 | 0.63 (0.37-1.08) | .1 |
| 3 | 0.77 (0.51-1.16) | .2 | 1.06 (0.62-1.79) | .8 | 1.21 (0.70-2.09) | .5 | 0.70 (0.40-1.25) | .2 | 0.63 (0.38-1.05) | .08 | 0.97 (0.59-1.60) | .9 | 0.63 (0.36-1.09) | .1 |
| 4 | 0.80 (0.52-1.25) | .3 | 1.11 (0.63-1.95) | .7 | 1.14 (0.64-2.05) | .7 | 0.73 (0.40-1.34) | .3 | 0.68 (0.39-1.19) | .2 | 0.87 (0.51-1.50) | .6 | 0.62 (0.35-1.11) | .1 |
| **Feeling nervous** | | | | | | | | | | | | | | |
| 0 | 0.60 (0.38-0.95) | .03 | 1.02 (0.58-1.77) | .9 | 0.55 (0.29-1.02) | .06 | 0.47 (0.23-0.96) | .04 | 0.86 (0.49-1.48) | .6 | 0.66 (0.36-1.20) | .2 | 0.99 (0.58-1.71) | .9 |
| 1 | 0.59 (0.38-0.93) | .02 | 1.01 (0.58-1.78) | .9 | 0.51 (0.27-0.96) | .04 | 0.46 (0.22-0.96) | .04 | 0.82 (0.47-1.44) | .5 | 0.63 (0.34-1.15) | .1 | 0.96 (0.56-1.67) | .9 |
| 2 | 0.56 (0.35-0.90) | .02 | 1.00 (0.57-1.77) | .9 | 0.49 (0.25-0.94) | .03 | 0.47 (0.23-0.98) | .05 | 0.85 (0.49-1.48) | .6 | 0.60 (0.32-1.14) | .1 | 0.85 (0.47-1.51) | .6 |
| 3 | 0.56 (0.35-0.90) | .02 | 1.02 (0.58-1.82) | .9 | 0.49 (0.25-0.94) | .03 | 0.49 (0.23-1.02) | .06 | 0.83 (0.47-1.45) | .5 | 0.60 (0.31-1.14) | .1 | 0.87 (0.48-1.57) | .6 |
| 4 | 0.58 (0.35-0.96) | .03 | 1.17 (0.64-2.14) | .6 | 0.50 (0.25-1.01) | .05 | 0.59 (0.28-1.27) | .2 | 0.89 (0.49-1.63) | .7 | 0.66 (0.32-1.36) | .3 | 0.83 (0.44-1.57) | .6 |
| **Difficulty falling asleep** | | | | | | | | | | | | | | |
| 0 | 1.04 (0.70-1.53) | .9 | 0.77 (0.47-1.28) | .3 | 1.26 (0.75-2.09) | .4 | 0.77 (0.45-1.32) | .3 | 0.76 (0.47-1.24) | .3 | 0.83 (0.52-1.34) | .5 | 1.01 (0.62-1.64) | .9 |
| 1 | 1.03 (0.70-1.52) | .9 | 0.76 (0.46-1.27) | .3 | 1.19 (0.71-2.00) | .5 | 0.76 (0.44-1.33) | .3 | 0.73 (0.45-1.20) | .2 | 0.80 (0.49-1.29) | .4 | 0.99 (0.61-1.62) | .9 |
| 2 | 1.05 (0.70-1.56) | .8 | 0.73 (0.44-1.22) | .2 | 1.18 (0.69-2.04) | .5 | 0.76 (0.44-1.33) | .3 | 0.72 (0.44-1.19) | .2 | 0.84 (0.51-1.37) | .5 | 0.99 (0.60-1.65) | .9 |
| 3 | 1.10 (0.74-1.65) | .6 | 0.77 (0.46-1.31) | .3 | 1.14 (0.66-1.97) | .6 | 0.78 (0.44-1.38) | .4 | 0.70 (0.42-1.16) | .2 | 0.80 (0.49-1.32) | .4 | 1.02 (0.61-1.71) | .9 |
| 4 | 1.20 (0.78-1.85) | .4 | 0.98 (0.56-1.71) | .9 | 1.27 (0.71-2.27) | .4 | 0.89 (0.49-1.59) | .7 | 0.70 (0.40-1.21) | .2 | 0.92 (0.54-1.58) | .8 | 1.04 (0.60-1.81) | .9 |
| **Difficulty staying asleep** | | | | | | | | | | | | | | |
| 0 | 1.09 (0.74-1.61) | .7 | 0.71 (0.43-1.17) | .2 | 1.04 (0.62-1.75) | .9 | 0.74 (0.43-1.25) | .3 | 0.90 (0.56-1.45) | .7 | 0.89 (0.56-1.42) | .6 | 0.99 (0.61-1.61) | .9 |
| 1 | 1.09 (0.74-1.60) | .7 | 0.71 (0.43-1.16) | .2 | 1.01 (0.60-1.71) | .9 | 0.73 (0.43-1.25) | .3 | 0.88 (0.54-1.42) | .6 | 0.88 (0.55-1.40) | .6 | 0.98 (0.60-1.60) | .9 |
| 2 | 1.09 (0.73-1.63) | .7 | 0.68 (0.42-1.13) | .1 | 0.93 (0.54-1.61) | .8 | 0.73 (0.43-1.26) | .3 | 0.86 (0.53-1.40) | .5 | 0.99 (0.61-1.60) | .9 | 0.96 (0.58-1.60) | .9 |
| 3 | 1.15 (0.77-1.72) | .5 | 0.71 (0.43-1.18) | .2 | 0.96 (0.55-1.66) | .9 | 0.74 (0.43-1.27) | .3 | 0.86 (0.53-1.41) | .6 | 1.05 (0.64-1.70) | .9 | 1.03 (0.62-1.73) | .9 |
| 4 | 1.01 (0.66-1.54) | .9 | 0.70 (0.40-1.20) | .2 | 0.91 (0.51-1.62) | .7 | 0.74 (0.42-1.30) | .3 | 0.75 (0.44-1.28) | .3 | 1.02 (0.61-1.72) | .9 | 0.93 (0.54-1.60) | .8 |
| **Feeling irritable** | | | | | | | | | | | | | | |
| 0 | 0.85 (0.55-1.30) | .4 | 0.84 (0.48-1.46) | .5 | 0.74 (0.42-1.33) | .3 | 0.49 (0.25-0.95) | .04 | 0.56 (0.32-0.97) | .04 | 0.65 (0.37-1.14) | .1 | 0.90 (0.50-1.59) | .7 |
| 1 | 0.83 (0.54-1.29) | .4 | 0.84 (0.47-1.47) | .5 | 0.72 (0.40-1.30) | .3 | 0.48 (0.24-0.97) | .04 | 0.52 (0.29-0.92) | .02 | 0.63 (0.36-1.11) | .1 | 0.85 (0.47-1.51) | .6 |
| 2 | 0.78 (0.49-1.23) | .3 | 0.81 (0.46-1.43) | .8 | 0.65 (0.35-1.21) | .2 | 0.47 (0.24-0.95) | .04 | 0.53 (0.30-0.94) | .03 | 0.69 (0.38-1.23) | .2 | 0.88 (0.48-1.59) | .7 |
| 3 | 0.76 (0.48-1.20) | .2 | 0.80 (0.45-1.43) | .8 | 0.66 (0.35-1.24) | .2 | 0.47 (0.23-0.94) | .03 | 0.52 (0.29-0.93) | .03 | 0.60 (0.33-1.09) | .1 | 0.88 (0.48-1.62) | .7 |
| 4 | 0.79 (0.48-1.30) | .4 | 0.70 (0.37-1.33) | .3 | 0.73 (0.38-1.41) | .4 | 0.51 (0.24-1.06) | .07 | 0.57 (0.30-1.06) | .08 | 0.66 (0.35-1.26) | .2 | 0.93 (0.48-1.80) | .9 |
| **Feeling sad** | | | | | | | | | | | | | | |
| 0 | 0.63 (0.42-0.96) | .03 | 0.66 (0.39-1.13) | .1 | 0.88 (0.51-1.52) | .6 | 0.65 (0.36-1.15) | .1 | 0.58 (0.34-0.99) | .05 | 0.49 (0.29-0.84) | .01 | 1.11 (0.67-1.84) | .7 |
| 1 | 0.60 (0.40-0.92) | .02 | 0.65 (0.38-1.12) | .1 | 0.79 (0.45-1.38) | .4 | 0.60 (0.33-1.08) | .09 | 0.54 (0.31-0.93) | .03 | 0.47 (0.27-0.81) | .006 | 1.05 (0.63-1.76) | .8 |
| 2 | 0.59 (0.38-0.91) | .02 | 0.61 (0.35-1.06) | .08 | 0.76 (0.42-1.35) | .3 | 0.60 (0.33-1.08) | .09 | 0.53 (0.31-0.93) | .03 | 0.47 (0.27-0.83) | .009 | 1.01 (0.60-1.73) | .9 |
| 3 | 0.60 (0.39-0.93) | .02 | 0.61 (0.35-1.07) | .09 | 0.81 (0.45-1.46) | .5 | 0.60 (0.33-1.09) | .09 | 0.55 (0.32-0.96) | .04 | 0.45 (0.25-0.79) | .006 | 1.03 (0.60-1.77) | .9 |
| 4 | 0.66 (0.41-1.06) | .08 | 0.68 (0.37-1.23) | .2 | 0.85 (0.46-1.58) | .6 | 0.58 (0.31-1.09) | .09 | 0.56 (0.31-1.03) | .06 | 0.46 (0.25-0.86) | .01 | 1.17 (0.66-2.07) | .6 |
| **Feeling anxious** | | | | | | | | | | | | | | |
| 0 | 0.81 (0.50-1.32) | .4 | 0.84 (0.45-1.55) | .6 | 0.78 (0.39-1.55) | .5 | 0.27 (0.11-0.66) | .004 | 0.46 (0.23-0.95) | .04 | 0.80 (0.44-1.48) | .5 | 1.26 (0.70-2.27) | .4 |
| 1 | 0.79 (0.48-1.28) | .3 | 0.82 (0.44-1.54) | .5 | 0.72 (0.36-1.45) | .4 | 0.25 (0.10-0.60) | .002 | 0.43 (0.21-0.89) | .02 | 0.76 (0.41-1.40) | .4 | 1.19 (0.66-2.16) | .6 |
| 2 | 0.79 (0.48-1.31) | .4 | 0.80 (0.43-1.51) | .5 | 0.61 (0.29-1.30) | .2 | 0.24 (0.10-0.60) | .002 | 0.43 (0.21-0.89) | .02 | 0.87 (0.47-1.63) | .7 | 1.01 (0.54-1.88) | .9 |
| 3 | 0.80 (0.49-1.34) | .4 | 0.89 (0.47-1.70) | .7 | 0.66 (0.31-1.42) | .3 | 0.25 (0.10-0.62) | .003 | 0.41 (0.20-0.86) | .02 | 0.79 (0.42-1.50) | .8 | 0.96 (0.51-1.80) | .9 |
| 4 | 0.96 (0.56-1.63) | .9 | 0.88 (0.44-1.75) | .7 | 0.66 (0.29-1.48) | .3 | 0.30 (0.12-0.75) | .01 | 0.45 (0.21-0.98) | .05 | 0.81 (0.41-1.60) | .8 | 0.92 (0.46-1.82) | .8 |
| Model 0: crude analysis.  Model 1: age, sex.  Model 2: age, sex, psychiatric treatment.  Model 3: age, sex, psychiatric treatment, acute start dialysis.  Model 4: age, sex, psychiatric treatment, acute start dialysis, employment status, living situation. | | | | | | | | | | | | | | |

**Supplementary Figure 1. Study flowchart.**

**
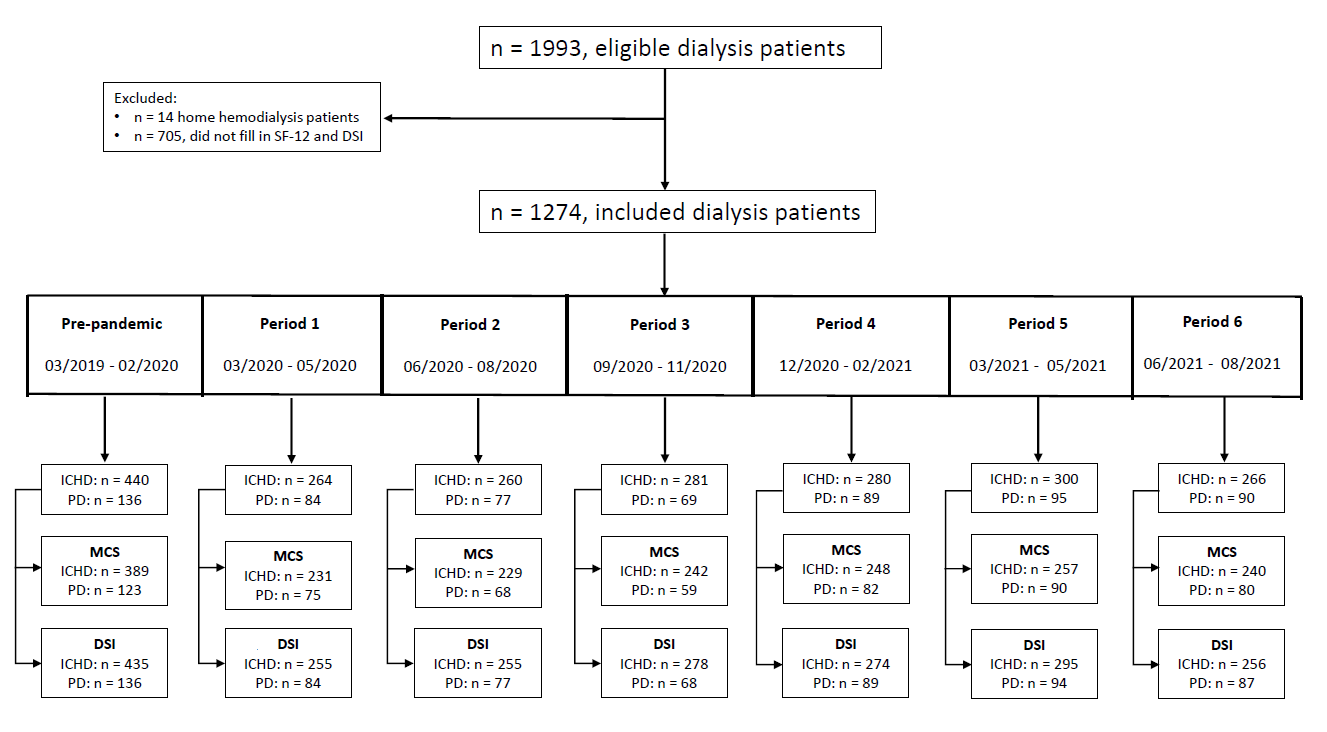
**
